# The effect of order of dwells on the first dwell gaze bias for eventually chosen items

**DOI:** 10.1371/journal.pone.0181641

**Published:** 2017-07-19

**Authors:** Takuya Onuma, Yuwadee Penwannakul, Jun Fuchimoto, Nobuyuki Sakai

**Affiliations:** 1 Department of Psychology, Graduate School of Arts and Letters, Tohoku University, Sendai, Miyagi, Japan; 2 Division for Interdisciplinary Advanced Research and Education, Tohoku University, Sendai Miyagi, Japan; Technion Israel Institute of Technology, ISRAEL

## Abstract

The relationship between choice and eye movement has gained marked interest. The gaze bias effect, i.e., the tendency to look longer at items that are eventually chosen, has been shown to occur in the first dwell (initial cohesion of fixations for an item). In the two-alternative forced-choice (2AFC) paradigm, participants would look at one of the items first (defined as *first look*; FL), and they would then move and look at another item (*second look*; SL). This study investigated how the order in which the chosen items were looked at modulates the first dwell gaze bias effect. Participants were asked to assert their preferences and perceptual 2AFC decisions about human faces (Experiment 1) and daily consumer products (Experiment 2), while their eye movements were recorded. The results showed that the first dwell gaze bias was found only when the eventually chosen item was looked at after another one; the chosen item was looked at for longer as compared to the not-chosen item in the SL, but not in the FL. These results indicate that participants actively allocate more time to looking at a subsequently chosen item only after they perceive both items in the SL. Therefore, the selective encoding seems to occur in the early comparison stage of visual decision making, and not in the initial encoding stage. These findings provide insight into the relationship between choice and eye movement.

## Introduction

We constantly make many choices in everyday life. Whether in front of a supermarket shelf or on mobile devices (e.g., smartphones and laptops), choices are mainly based on visual cues (e.g., package design, price tag, and advertisement) related to the products [1]; thus, visual attention plays an important role in our daily choices. Consequently, interest in eye movement monitoring has recently grown, because tracking of visual attention allows speculation on the psychological events that occur prior to explicit choice (such as cognitive stages or processes), with a millisecond time-resolution [2, 3].

Shimojo and his colleagues have investigated the role of eye movements in visual decision making [4–6]. Using images of human faces as visual stimuli, they found that participants looked at items that were eventually chosen significantly more often as compared to not-chosen items in the preference task, but not in other perceptual-decision tasks. This suggested that eye movements not only reflect, but also influence preferences through a positive-feedback loop; people tend to look longer at items that they like (i.e., preferential looking [7]) and tend to like items that they look at for a longer duration (i.e., mere exposure effect [8]). This gaze cascade effect is thought to be an example of the down-stream effects of visual attention; i.e., more attention leads to greater preference for the item [3].

However, recent studies have challenged Shimojo and his colleagues’ model, particularly in terms of task specificity and the time course of the gaze bias effect. Glaholt and Reingold [9, 10], using photos including natural scenes, etc., tested this model with two different decision tasks; preference and perceptual-decision tasks. Their results showed that participants looked at chosen items more often than at not-chosen items, not only in preference tasks, but also in perceptual-decision tasks. Therefore, it has been suggested that, rather than implying causality of preference formation, the gaze bias effect reflects a general aspect of visual decision making, such as the top-down control of visual attention to the most informative item for the current task. Furthermore, using dwell-based analyses, Glaholt and Reingold [9, 10] identified the gaze bias during the first dwell time; the amount of time spent looking at items that were eventually chosen before first leaving it was longer than that spent looking at not-chosen items. To account for these results, Glaholt and Reingold [9, 10] suggested that people engage actively in selective encoding―actively evaluating items during encoding and excluding items that do not fit the given criteria. They also suggested that this top-down control of visual attention results in the allocation of more time to looking at eventually chosen items than to looking at not-chosen items, even in the early stage of decision making. More recently, Schotter, Berry, McKenzie, and Rayner [11] replicated the findings of Glaholt and Reingold [9, 10], and Schotter, Gerety, and Rayner [12] reported that participants may have known initially which item they were going to choose, even if they did not know what the alternatives were.

**Fig 1:**
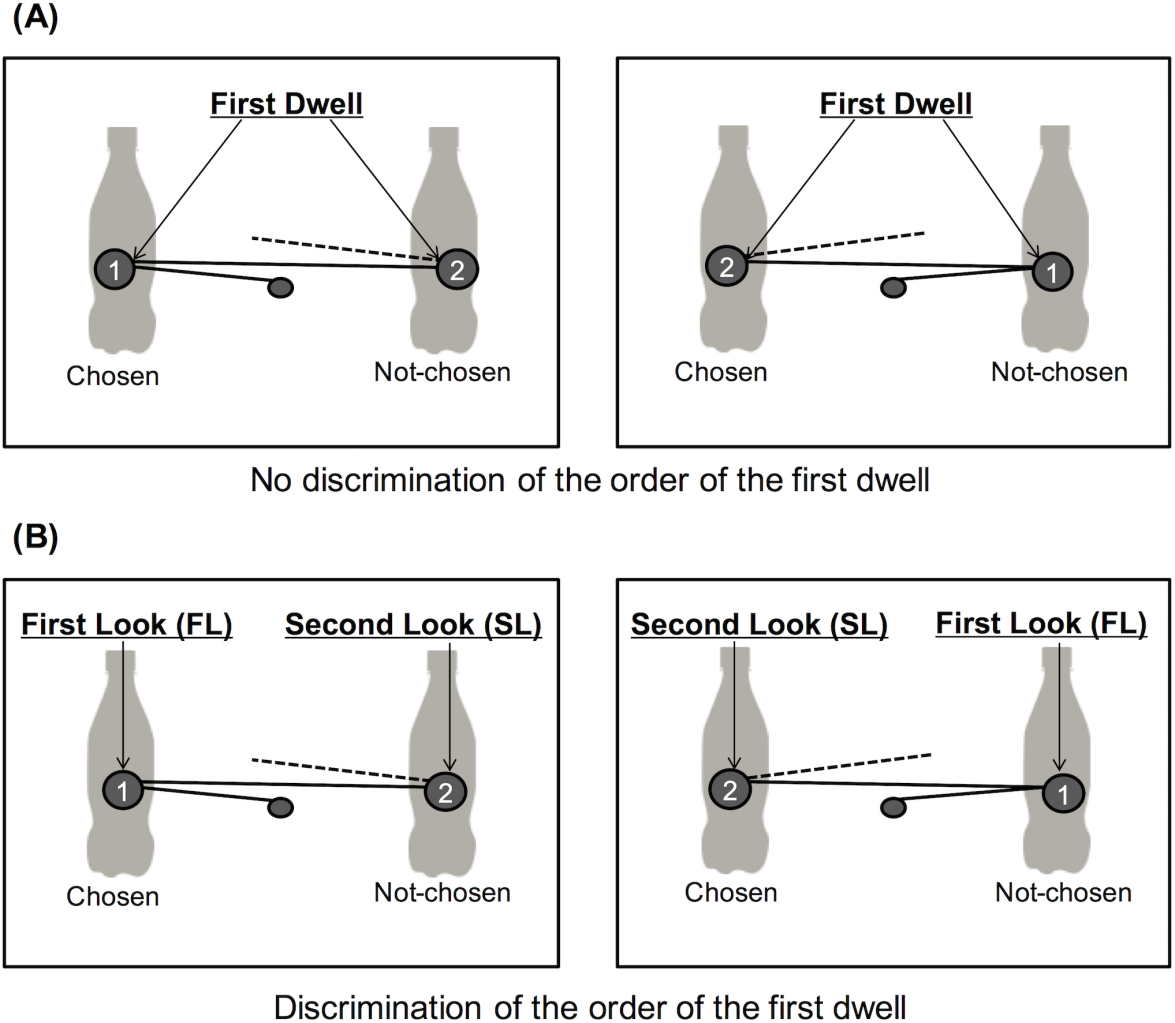
**Different approaches to first dwell time of (A) previous studies [11, 12] and (B) the present study, with the 2AFC paradigm.** Dots and solid lines represent participants’ eye movement, and the numbers represent the order of dwells. Dashed lines represent eye movements after the second look (not argued in this paper). In the previous studies, there was no discrimination between the first look (FL) and the second look (SL), because the effects of the order of dwells were not considered. In the present study, the FL and SL were clearly discriminated and the relationship between the order of dwells and gaze bias was considered.

Therefore, it was thought that it is possible to predict consumer choice by the dwell-based analysis of the first dwell time, even before choices are made explicitly. However, the analysis of first dwell time conducted in previous studies [11, 12] requires further discussion. These authors measured the amount of time spent on looking at an item (chosen and not-chosen) before first leaving it, and defined this index as the “first dwell time,” but they did not take the order of dwells into account (Fig 1A). In the two-alternative forced-choice (2AFC) paradigm, participants’ eyes would dwell on one of the items first (we defined this dwell as the *first look*; FL), and then they move to and dwell at another item (the *second look*; SL). In some cases, the chosen item would be dwelled at during the FL and the not-chosen item during the SL, and *vice versa* (Fig 1B). This is crucial, because these two types of dwells would involve different stages of decision making. Recent neurophysiological studies [13–15] suggested that decision making involves a two-stage mechanism in which items are first encoded and evaluated individually (the encoding stage), and they are then compared to yield a choice (the comparison stage). In the 2AFC paradigm, at the time of the FL, only encoding of the current item would occur; because, in that time, participants appear not to know about another item. On the other hand, at the time of the SL, encoding of the current item as well as comparison with another item would occur, because participants have already encoded another item during the FL and stored its visual representation. If the FL involves only the encoding stage, but the SL involves both encoding and comparison stages, selective encoding (and the gaze bias as an outcome) would only occur in the SL, because until that comparison stage, participants cannot know which one of the items is the more appropriate choice.

In fact, Lindsen, Jones, Shimojo, and Bhattacharya [16], using electroencephalogram (EEG) recordings during 2AFC tasks, showed the interactive effects between choice and look order on EEG components. In their study, participants were asked to make preference decisions between two faces. To manipulate the order of the participants’ looking behavior, two faces were presented sequentially on a monitor; i.e., one face was presented alone, and was then replaced by another face. The viewing duration of both faces was unrestricted and fully controlled by the participants. In this case, looking at the face presented first (or second) corresponded to the FL (or SL). EEG data following the onset of the face presented second showed that frontal theta band activity, which is associated with working memory load [17], increased when this second face was preferred (i.e., the chosen item was presented in the SL). In contrast, posterior gamma band oscillation was associated with reactivation of visual representation and increased memory retrieval [18] when the face presented first was preferred (i.e., the chosen item was presented in the FL). However, these effects were not found following the onset of the first face presentation. Thus, these results also indicated that the FL involves only the encoding stage, but the SL involves both encoding and comparison stages.

**Fig 2:**
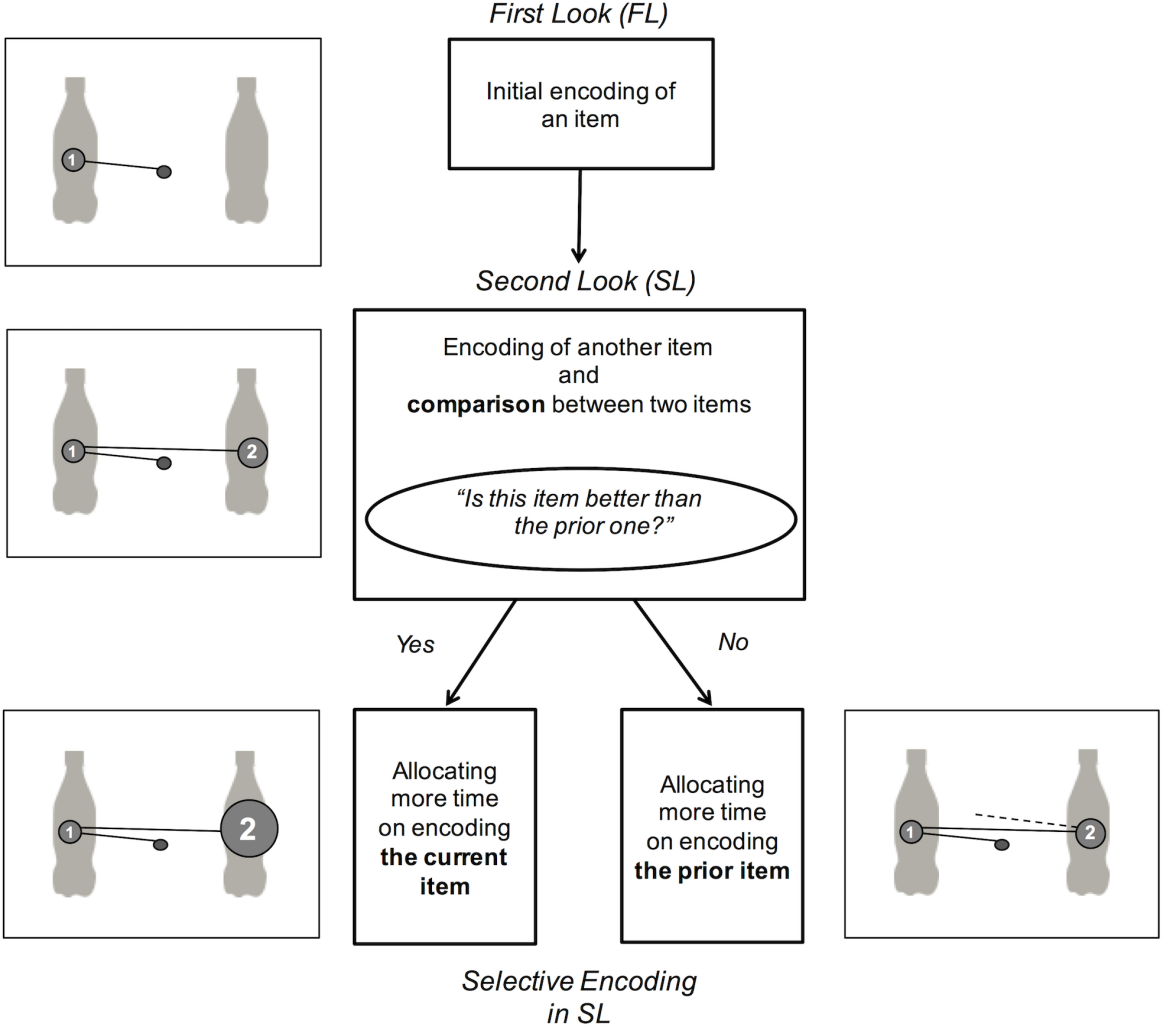
A schematic representation of selective encoding in the 2AFC paradigm suggested and examined in the present study. At the time of the first look (FL) (i.e., initial encoding stage), participants cannot know whether an item is more appropriate than another item; thus, selective encoding does not occur. On the other hand, at the time of the second look (SL) (i.e., encoding and comparison stage), participants know all the items presented and they can judge which item meets their decision criteria better. Therefore, selective encoding occurs only in the SL, and not in the FL.

Based on this recent evidence, we proposed a modified model of selective encoding in the 2AFC paradigm (Fig 2) and examined this model in the subsequent experiments. In the beginning of a trial, two items are simultaneously presented on the monitor. First, the participants look at an item, either the one on the left or the one on the right. During this time (FL), the participants do not know which they will choose, and only encoding of the current item occurs. Next, the participants look at another item, which is then encoded. During this time (SL), the participants have come to know about both the items for the first time and they can now compare between the current item and the visual representation of the previous item (“Does this item better meet my decision-making criteria than the previous one?”). When the current item is considered better than the previous item (i.e., the item in the SL is eventually chosen), the participants allocate more time to further encoding the item that they are looking at currently. On the other hand, when the current item is not considered more appealing than the previous item, the participants cease looking at the item immediately and allocate more time to further encoding the first item. In this model, selective encoding is not thought to occur throughout the first dwells (FL and SL). Rather, selective encoding is considered to occur only after the beginning of the early comparison stage (SL).

The primary purpose of this study was to examine these hypotheses according to our model of selective encoding. First, the FL reflects only the initial encoding stage, whereas the SL reflects both the early encoding and comparison stages. Thus, we hypothesized that the SL would take more time than the FL would. Second, until the time of the SL, participants cannot compare the items and do not know which one of the items would be more appropriate to choose. Therefore, we hypothesized that selective encoding (and gaze bias for eventually chosen items) would be found only in the SL and not in the FL; thus, dwell time would be longer for the chosen items than for the not-chosen items, only in the SL and not in the FL.

Throughout our two experiments, we asked participants to make 2AFCs with two different decision prompts: preference and perceptual-decision tasks. Previous studies have suggested that selective encoding reflects a general aspect of visual decision making [9–12]; therefore, we hypothesized that there would be no difference in gaze bias between the preference and perceptual tasks. In Experiment 1, we asked participants to make preference and roundness decisions on human faces and recorded participants’ eye movements during the decision making. To replicate the findings in the consumer context, we asked participants to make preference and brightness decisions on commercial food products (wine and snacks) in Experiment 2.

## Experiment 1

### Material and methods

#### Participants

Twenty-one university students (10 males and 11 females, *M*_*age*_ = 22.10) with normal or corrected-to-normal vision participated in this experiment. Note that 10 of 21 participants were added after the analysis of the data from first 12 participants. These two sets of data were treated together since our pilot analysis found no significant difference between first dataset and second dataset (see S1 Table).

#### Ethics statement

An explanation about the experiment was given to the participants and written informed consent was obtained from them. This experiment was conducted based on the ethics guidelines and was approved by the Ethics Committee of the Graduate School of Arts and Letters, Tohoku University.

#### Apparatus

Eye movements were recorded via an Eye Tech TM3 infrared eye-tracker (30 Hz, EyeTech Digital Systems, Mesa, AZ, USA) and analyzed with QG-Plus (DITECT, Tokyo, Japan). Visual stimuli were presented on a 17-inch Iiyama ProLite E1706S monitor with a pixel resolution of 1280 × 1024, and participants were seated approximately 57 cm away from the monitor. Presentation of stimuli and recording of participants’ decisions were conducted with E-prime 2.0 (Psychology Software Tools, Sharpsburg, PA, USA).

#### Stimuli and design

Six photos of unknown human faces (3 men and 3 women, all Japanese) from the ATR database (ATR-Promotions, Osaka, Japan) were used as visual stimuli. All photos were collected from the database of emotionally neutral expressions. All photos were resized to 3.9 × 5.3° of the visual angle. Photos were presented in pairs so that participants could make 2AFC decisions. Totally 30 pairs were obtained where each of the 6 photos was presented 10 times, with different partners, at different (left or right) positions. The gap between the photos in a pair was about 7° of the visual angle. Although there may seem to be few stimuli and choice sets in this experiment, a previous study [19] with a similar choice-set size (32 choice sets) yielded valid and reliable results on the relationship between consumer preference choices and eye movement. Therefore, we considered that our set size was sufficient for detecting reliable results.

The experiment consisted of two task blocks: *preference task* (“which face do you prefer?”) and *roundness task* (“which face is rounder?”). Each block consisted of 30 pairs of photos as described above (i.e., 30 trials in each task block); therefore, each participant made 60 decisions in total. The order of the tasks was counter-balanced across participants, and the order of pairs was randomized. Each pair of photos was presented on a white background with one photo on the left side of the screen and the other on the right.

#### Procedure

**Fig 3:**
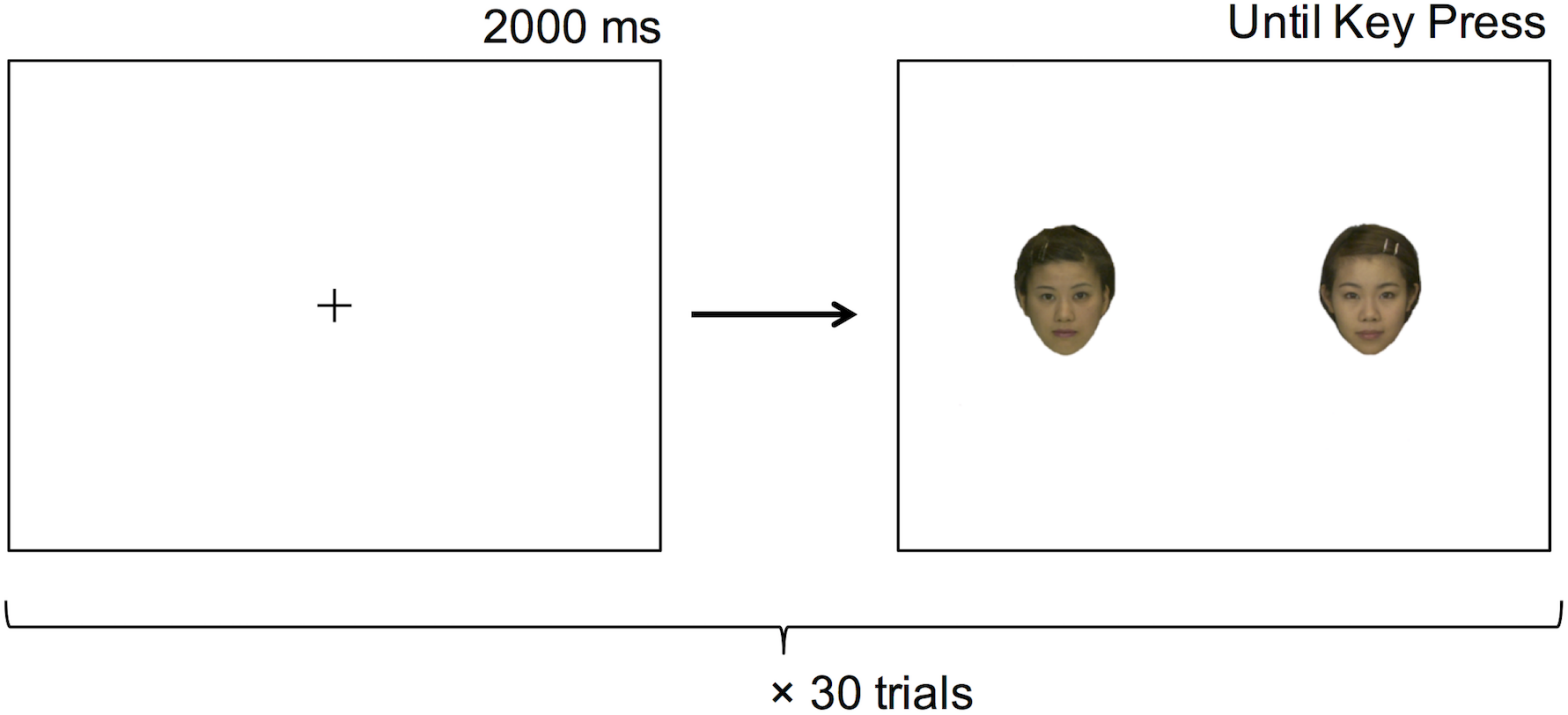
Example of a trial in Experiment 1. After a fixation point was presented in the center of the screen (2000 ms), a pair of photos was presented on the monitor. Once participants made a decision by pressing a key, the photos were replaced by the fixation point, before starting the next trial. This procedure was repeated for 30 trials in each task block (preference and roundness tasks).

At the beginning of each block, instructions for the experiment were shown on the monitor. Participants read the instructions at their own pace. At the beginning of each trial (Fig 3), a fixation point was presented in the center of the screen (2000 ms). Then, a pair of photos was presented on the screen. Participants had been instructed to look at the photos freely, in any order and any number of times, and that, once they had made a decision, they should press the key indicating their choice. Participants pressed the “1” key to choose the photo on the left or the “3” key to choose the photo on the right. Once participants had made a decision, the pair of photos was replaced by a fixation point, thereby starting the next trial. This procedure was repeated for 30 trials in each task block.

#### Analyses

Based on the recorded eye movement data, we calculated first dwell time (ms) for each item, which was the time spent looking at an item before first leaving it. Our data comprised 2,520 observations (21 participants × 2 tasks × 30 trials × 2 items). Following the method used in a previous study [12], we excluded trials if no valid dwell was recorded or if participants looked at only one of the items (1.5% of the data). The distribution of the dwell time was highly skewed (skewness = 2.04), thus skew correction was performed by the log transformation (skewness = -0.58) and was then used for the analysis.

Generalized linear mixed models (GLMMs) were used to determine the impact of various factors on first dwell time. One of the merits of GLMMs is that they can manage nested data, for instance, one trial includes two dwell data points (i.e., for chosen and not-chosen items) in this study. Additionally, they can include not only fixed effects, but also random effects, such as different response tendencies among the participants. In fact, GLMMs have been used in recent eye-tracking studies [20–22]. For the obtained data, we used the function *lmer* of the *lmerTest* package available in *R* [23].

We conducted the GLMM analyses based on our own model of selective encoding, which included “Task” (decision tasks: preference vs. roundness), “Look” (the order of the dwells for the item: FL vs. SL) and “Choice” (decision consequence for the item: chosen vs. not-chosen) as a fixed-effect of the first dwell time. These fixed-effects were converted into binary data; preference (0) and roundness (1) for “Task,” FL (0) and SL (1) for “Look,” and chosen (0) and not-chosen (1) for “Choice.” To examine the potential effect of repetitive presentation of stimuli, “Trial” (the order of the trials in each task block) was also included as a fixed-effect. Then, these fixed-effects were centered to create a mean of 0. This model also included “Participant” (participant ID) as a random effect to take into account different response tendencies and different sensitivity for the fixed-effects among participants. Thus, this model included the random effect of “Participant” for the intercept and slope for each fixed-effect. This “maximal random effects structure” is suggested to be the best way to conduct confirmatory hypothesis testing [24].

For the GLMMs, we report regression coefficients (*b*s), which estimate the effect size, standard errors (SEs), and *t*-values (*t*s). While the GLMMs were applied to whole log-transformed observations, the means reported in the results section and figures were calculated from participant averages of the raw dwell time (ms) [12]. We hypothesized that the first dwell time for eventually chosen items would be greater than that for not-chosen items only in the SL, but not in the FL; i.e., there would be a significant fixed-effect of interaction between “Look” and “Choice.”

### Results

**Fig 4:**
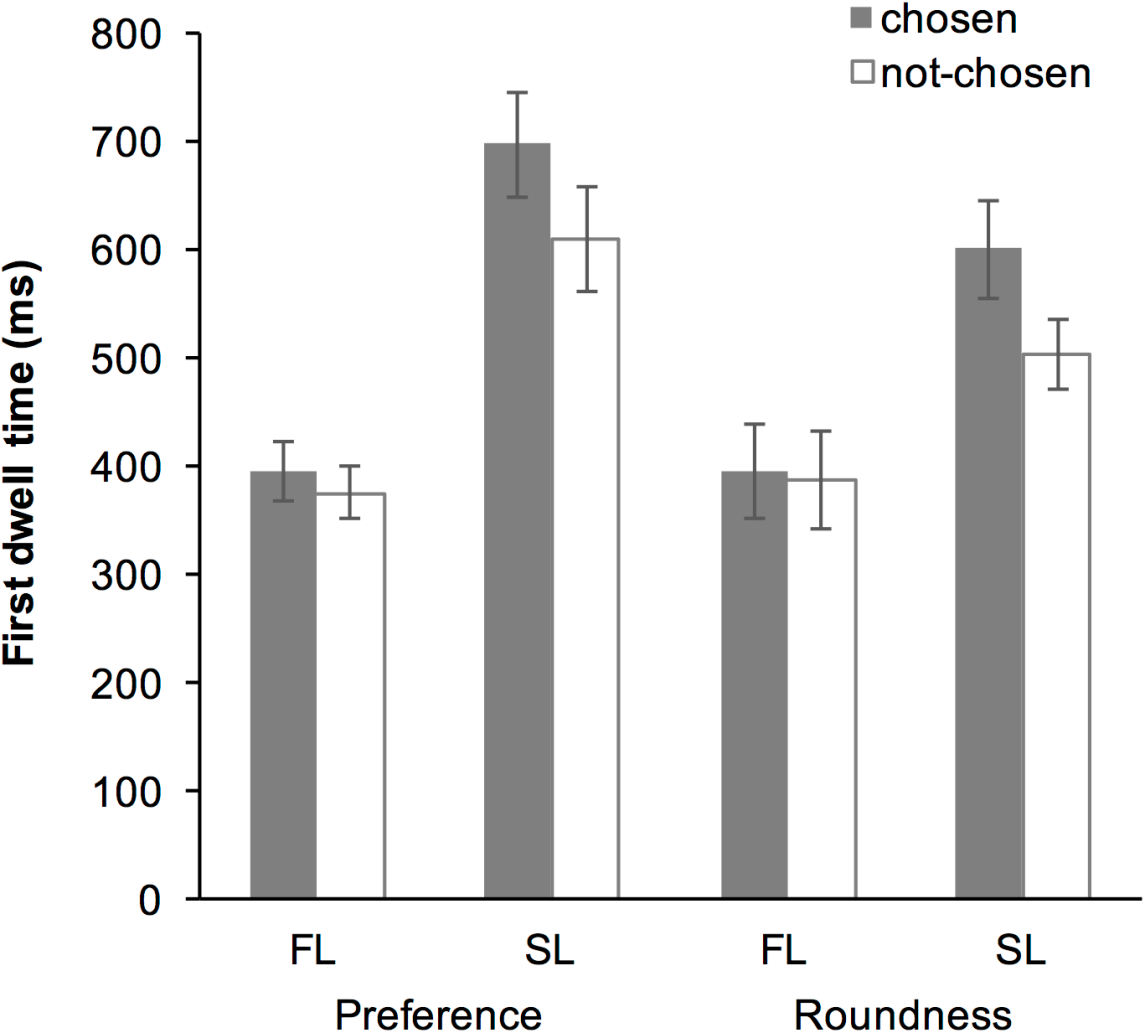
Averaged first dwell time in Experiment 1. Error bars represent standard error of the mean.

The averaged data suggested that the first dwell time in the SL was greater than that in the FL, and that first dwell time was greater for the chosen item than for the not-chosen item in the SL, but not in the FL (Fig 4). The GLMM revealed that the fixed-effect of “Task,” “Look,” “Choice,” interaction between “Task” and “Look,” and interaction between “Look” and “Choice” were all significant (Table 1). *Post hoc* analysis of the interaction between “Task” and “Look” revealed that the fixed-effect of “Look” was significant both in the preference subset (*b* = 0.530, SE = 0.055, *t* = 9.56, *p* < 0.001) and in the roundness subset (*b* = 0.402, SE = 0.046, *t* = 8.670, *p* < 0.001); the first dwell time of the SL was greater than that of the FL in both tasks. The analysis also revealed that, while the fixed-effect of “Task” was significant in the SL subset (*b* = -0.151, SE = 0.032, *t* = -4.690, *p* < 0.001), it was not in the FL subset (*b* = -0.017, SE = 0.046, *t* = -0.380, *p* = 0.704); the first dwell time of the SL in the preference task was greater than that in the roundness task.

**Table 1:** Generalized linear mixed models fitting the first dwell time in Experiment 1.

	Fixed effects				Random effects (variance)
Predictor	*b*	SE	*t*		By-participants
Intercept	6.029	0.059	101.560	***	0.072
Task	-0.084	0.029	-2.880	**	0.010
Look	0.465	0.042	11.050	***	0.030
Choice	-0.104	0.022	-4.730	***	0.003
Trial	-0.002	0.002	-0.810		0.000
Task×Look	-0.129	0.045	-2.860	**	0.013
Task×Choice	-0.029	0.048	-0.600		0.018
Look×Choice	-0.112	0.046	-2.430	*	0.014
Task×Trial	0.005	0.004	1.270		0.000
Look×Trial	-0.004	0.003	-1.240		0.000
Choice×Trial	0.004	0.003	1.370		0.000
Task×Look×Choice	-0.028	0.107	-0.260		0.117
Task×Look×Trial	-0.009	0.007	-1.290		0.001
Task×Choice×Trial	0.001	0.005	0.210		0.000
Look×Choice×Trial	-0.003	0.006	-0.570		0.000
Task×Look×Choice×Trial	-0.003	0.013	-0.190		0.002

“×” means an interaction between the predictors.

* *p* < 0.05

** *p* < 0.01

*** *p* < 0.001

*Post hoc* analysis of the interaction between “Look” and “Choice” revealed that the fixed-effect of “Look” was significant both in the chosen subset (*b* = 0.520, SE = 0.050, *t* = 10.45, *p* < 0.001) and in the not-chosen subset (*b* = 0.411, SE = 0.052, *t* = 7.930, *p* < 0.001); the first dwell time of the SL was greater than that of the FL. The analysis also revealed that while the fixed-effect of “Choice” was not significant in the FL subset (*b* = -0.047, SE = 0.027, *t* = -1.720, *p* = 0.086), but it was significant in the SL subset (*b* = -0.159, SE = 0.033, *t* = -4.820, *p* < 0.001); the first dwell time for the chosen item was greater than for the not-chosen item in the SL.

### Discussion

In this experiment, a significant fixed-effect of interaction between “Look” and “Choice” was found. *Post hoc* analysis revealed that the first dwell time of the SL was greater than that of the FL. This result supported our hypothesis that the SL requires more time than the FL does, because the SL reflects both the encoding and the comparison stages of the decision-making process [16]. Moreover, it was also revealed that the first dwell time for the eventually chosen item was greater than that for the not-chosen item in the SL, but the same was not observed in the FL. This result also supported our hypothesis that the selective encoding is stage-specific. In the FL, participants did not yet know the other item, and therefore, they could not judge whether they would choose that item. On the other hand, in the SL, participants knew both items and they could judge which item they would choose. Therefore, gaze bias for the eventually chosen item was present only in the SL and not in the FL.

The result also showed no differences in the gaze bias effect between the preference and perceptual-decision tasks, indicating that selective encoding reflects a general aspect of visual decision making [9–12]. On the other hand, a significant fixed-effect of interaction between “Task” and “Look” was found; the first dwell time of the SL in the preference task was greater than that in the roundness task. Although it is difficult to interpret the meaning of this difference, it is speculated that cognitive demands for initial comparison might be different between preference and non-preference task. Nevertheless, this possible difference between the preference and the non-preference tasks had no effect on selective encoding and the gaze bias.

Taken together, these results indicated that participants actively evaluated an item that matched their decision criteria and looked at the item longer only after other items were encoded. Selective encoding and gaze bias may therefore occur in the early comparison stage (SL), but not in the initial encoding stage (FL). To replicate the findings in other contexts, we asked participants in Experiment 2 to make preference and brightness decisions on commercial food products (wine and snacks). Selective encoding and gaze bias would reflect the general aspects of the decision-making process; thus, we hypothesized that commercial food products would be prone to a similar gaze bias found in Experiment 1.

## Experiment 2

### Material and methods

#### Participants

Twenty-two university students (11 males and 11 females, *M*_*age*_ = 22.52) with normal or corrected-to-normal vision participated in the experiment. As in Experiment 1, 11 of 22 participants were added after the analysis of the data from first 11 participants. These two sets of data were treated together since our pilot analysis found no significant difference between first dataset and second dataset (see S2 and S3 Tables).

#### Apparatus

Apparatus and recording methods were identical to those in Experiment 1.

#### Stimuli and design

The experiment consisted of two blocks: the *wine block* and the *snack block*. Six photos of bottles of red wine (wine block) and 6 photos of packages of snacks (snack block) were used as visual stimuli. Both products were available in Japanese markets. Each photo was resized to the same size (wine block, 5.3× 18.3° of the visual angle; snack block, 8.7× 11.6° of the visual angle). In each block, the photos were presented in pairs so that participants had to make 2AFC decisions. The gap between the photos in a pair was about 5° of the visual angle.

Each block consisted of two decision tasks: *preference task* (“which one do you prefer?”) and *brightness task* (“which one is brighter?”). The order of the tasks was a within-participant variable. Each task consisted of 30 pairs (choice sets) of photos made up from the 6 photos, by varying the position of the photos. Thus, each participant made a total of 60 decisions per block. Stimuli were presented in the same manner as in Experiment 1. The order of stimuli was randomized; randomization in task blocks was a within-participant variable.

#### Procedure

Procedure and instructions were identical to those in Experiment 1, except for the stimuli used.

#### Analyses

As in Experiment 1, we calculated first dwell time. Our data comprised 2,640 observations (22 participants × 2 tasks × 30 trials × 2 items) in the wine and snack blocks, but 8.1% of the observation in the wine block and 10.6% of those in the snack block were excluded following the criteria [12]. The distribution of the dwell time was highly skewed (3.12 in the wine block; 4.03 in the snack block), thus skew correction was performed by log transformation (-0.42 in the wine block; -0.45 in the snack block) and the data were then used for the analysis. GLMMs were used to determine the impact of various factors on first dwell time.

### Results

#### Wine block

**Fig 5:**
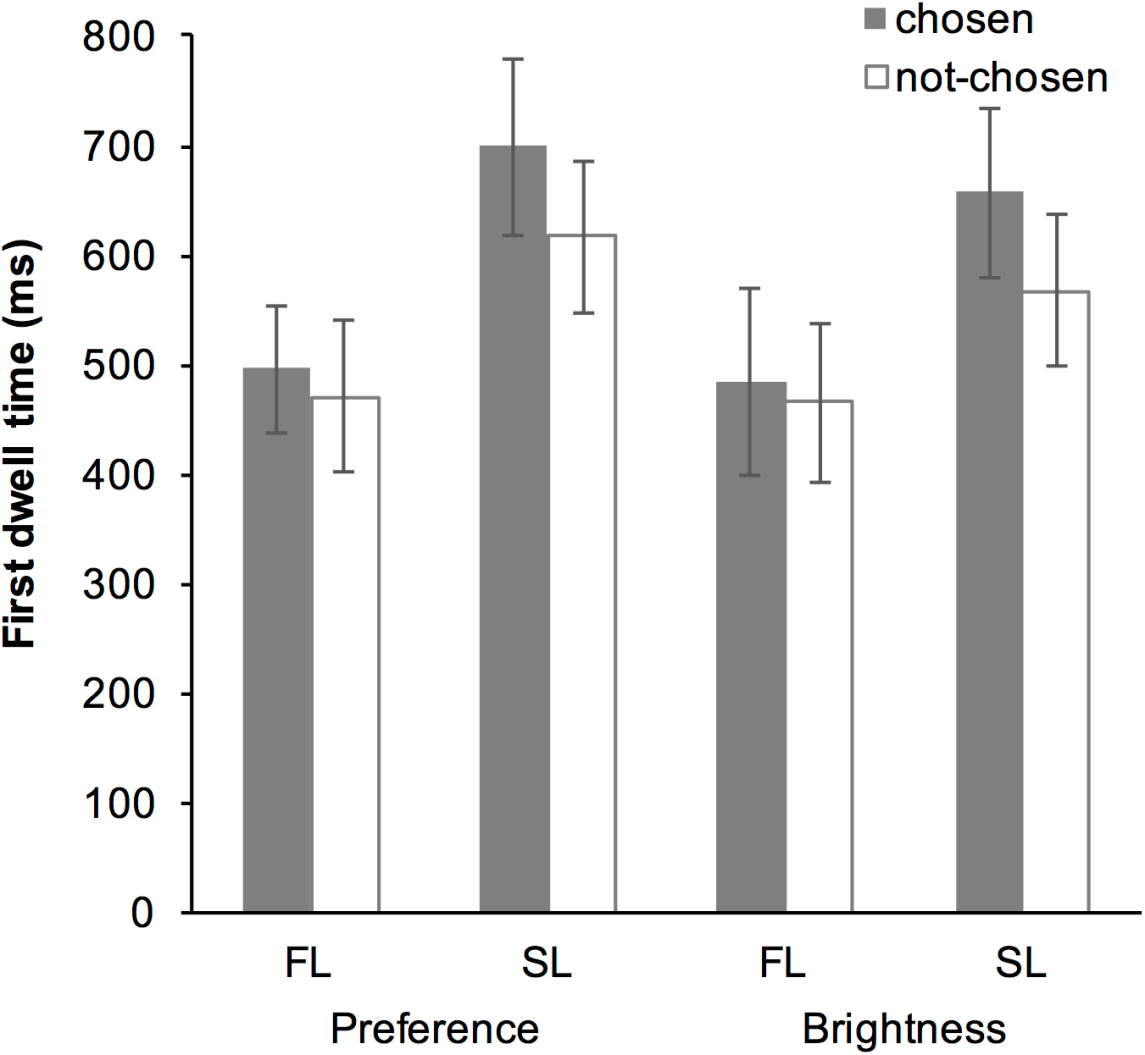
Averaged first dwell time in the wine block in Experiment 2. Error bars represent the standard error of the mean.

The averaged data suggested that the first dwell time in the SL was greater than that in the FL. Moreover, the data suggested that first dwell time was greater for the chosen item than for the not-chosen item in the SL, but the same was not observed in the FL (Fig 5). The GLMM highlighted the fixed-effect of “Look,” “Choice” and the interaction between “Look” and “Choice” (Table 2). The fixed-effect of “Trial” was also significant indicating that the first dwell time decreased as the trials progressed.

**Table 2:** Generalized linear mixed models fitting the first dwell time in the wine block in Experiment 2.

	Fixed effects				Random effects (variance)
Predictor	*b*	SE	*t*		By-participants
Intercept	6.023	0.091	65.900	***	0.180
Task	-0.054	0.078	-0.690		0.118
Look	0.303	0.055	5.510	***	0.052
Choice	-0.111	0.037	-2.980	**	0.016
Trial	-0.012	0.002	-4.930	***	0.000
Task×Look	-0.061	0.081	-0.750		0.085
Task×Choice	-0.031	0.150	-0.210		0.439
Look×Choice	-0.177	0.078	-2.280	*	0.072
Task×Trial	-0.001	0.004	-0.170		0.000
Look×Trial	-0.005	0.005	-1.010		0.000
Choice×Trial	0.004	0.004	1.010		0.000
Task×Look×Choice	-0.100	0.126	-0.790		0.112
Task×Look×Trial	-0.009	0.010	-0.960		0.001
Task×Choice×Trial	-0.003	0.008	-0.360		0.001
Look×Choice×Trial	-0.002	0.007	-0.210		0.000
Task×Look×Choice×Trial	-0.003	0.014	-0.240		0.001

“×” means an interaction between the predictors.

* *p* < 0.05

** *p* < 0.01

*** *p* < 0.001

*Post hoc* analysis of the interaction between “Look” and “Choice” revealed that the fixed-effect of “Look” was significant both in the chosen subset (*b* = 0.397, SE = 0.054, *t* = 7.390, *p* < 0.001) and in the not-chosen subset (*b* = 0.217, SE = 0.056, *t* = 3.850, *p* < 0.001); the first dwell time of the SL was greater than that of the FL. The analysis also revealed that while the fixed-effect of “Choice” was not significant in the FL subset (*b* = -0.021, SE = 0.040, *t* = -0.520, *p* = 0.603), but significant in the SL subset (*b* = -0.192, SE = 0.041, *t* = -4.740, *p* < 0.001); first dwell time for the chosen item was greater than for the not-chosen item in the SL.

#### Snack block

**Fig 6:**
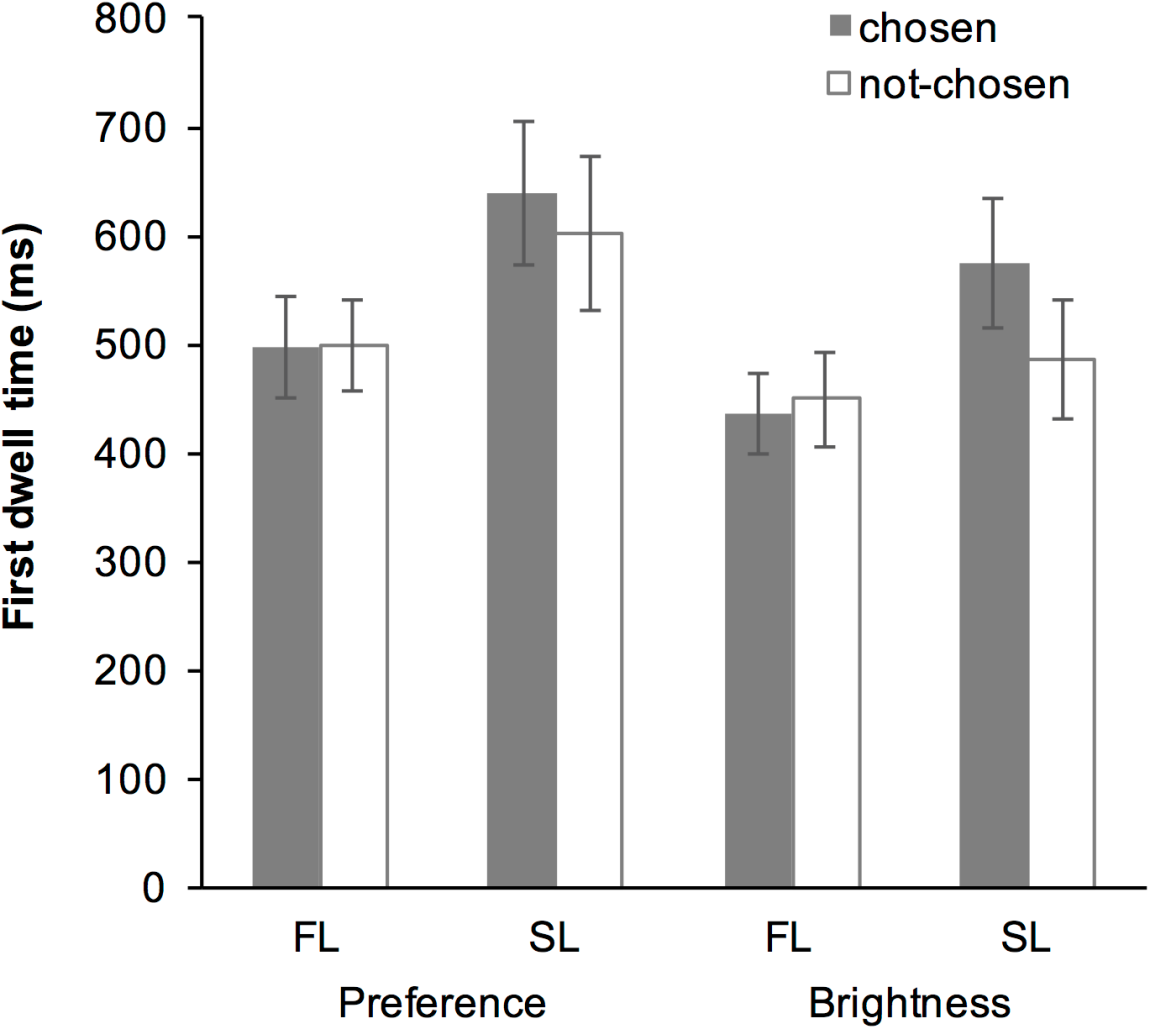
Averaged first dwell time in the snack block in Experiment 2. Error bars represent the standard error of the mean.

The averaged data suggested that the first dwell time in the SL was greater than that in the FL. Moreover, as shown in the wine block, the data suggested that first dwell time was greater for the chosen item than for the not-chosen item in the SL, but the same was not observed in the FL (Fig 6). The GLMM revealed that although the fixed-effect of “Task” and “Look” was significant, that of “Choice” (*p* = 0.114) was not and the interaction between “Look” and “Choice” (*p* = 0.093) was marginally significant (Table 3). The fixed-effect of “Trial” was also significant indicating that the first dwell time decreased as the trials progressed.

**Table 3:** Generalized linear mixed models fitting the first dwell time in the snack block in Experiment 2.

	Fixed effects				Random effects (variance)
Predictor	*b*	SE	*t*		By-participants
Intercept	6.000	0.105	57.380	***	0.237
Task	-0.138	0.062	-2.250	*	0.068
Look	0.165	0.056	2.950	**	0.054
Choice	-0.067	0.043	-1.580		0.025
Trial	-0.005	0.002	-2.690	**	0.000
Task×Look	-0.049	0.107	-0.460		0.192
Task×Choice	-0.021	0.080	-0.260		0.080
Look×Choice	-0.143	0.085	-1.680		0.099
Task×Trial	0.002	0.004	0.480		0.000
Look×Trial	-0.004	0.006	-0.690		0.001
Choice×Trial	-0.006	0.004	-1.660		0.000
Task×Look×Choice	-0.035	0.167	-0.210		0.369
Task×Look×Trial	0.004	0.010	0.360		0.002
Task×Choice×Trial	0.008	0.008	1.020		0.001
Look×Choice×Trial	0.008	0.007	1.140		0.000
Task×Look×Choice×Trial	0.027	0.015	1.780		0.002

“×” means an interaction between the predictors.

* *p* < 0.05

** *p* < 0.01

*** *p* < 0.001

### Discussion

Along with Experiment 1, in which the photos of human faces were used, the present results showed that the first dwell time of the SL was greater than that of the FL both in the wine and snack blocks. This result supported our hypothesis that the SL requires more time than the FL does, because the SL reflects both the encoding and the comparison stages of the decision-making process [16].

In the wine block, a significant fixed-effect of interaction between “Look” and “Choice” was found; the first dwell time for the eventually chosen item was greater than that for the not-chosen item in the SL, but not in the FL. Furthermore, there was no significant difference between the preference and perceptual tasks. These results further supported our hypothesis that the selective encoding reflects a general aspect of visual decision making, and that it occurs only in the comparison stage, not in the initial encoding stage of visual decision-making.

In the snack block, almost the same tendency was found, but fixed-effect of “Choice” was not significant and of interaction between “Look” and “Choice” was only marginally significant. One possible explanation for this marginal interaction is that prior knowledge regarding the snacks may have attenuated the selective encoding. Although we did not investigate this directly, the photos of wines presented in the wine block seemed to be unfamiliar to young Japanese participants, but those of snacks presented in the snack block seemed to be well-known to some participants. Previous consumer research showed that consumers tend to utilize prior knowledge to make more efficient choices [25, 26]. In such cases, the need to encode further information about the items is low. We speculated that since some participants had prior knowledge about the snack products and may have utilized it efficiently in the decision-making task, selective encoding and gaze bias was attenuated in the snack block.

In this experiment, 6 photos of wine or snack were repeatedly presented in each block. This repetitive stimulus presentation could lead participants to become familiar with the stimuli as the trials progresses. This point seems problematic since such familiarization within the experiment could also attenuate selective encoding and gaze bias. The result showed significant fixed-effect of “Trial” both in the wine and the snack blocks, indicating that the first dwell time decreased as the trials progressed. However, there was no significant interaction of “Trial” with “Look” and/or “Choice.” These results indicate that the repetitive stimulus presentation could make participants’ sampling behavior more effective through the progress of the experiment, but it did not modulate selective encoding and gaze bias.

## General discussion

Interest in explaining the relationship between choice and eye movement has grown in recent years [2, 3]. Previous studies have shown that, even at the start of choice tasks, a significant gaze bias exists, indicating selective encoding for eventually chosen items [9–12]. However, the previous model did not take the order of the first dwells (FL and SL) into account (Fig 1). There is evidence indicating that the FL and the SL involves different stages of visual decision making [16]. Therefore, we here proposed a modified model of selective encoding in the 2AFC paradigm (Fig 2), which included the order of the first dwells, and examined the model both in cognitive or social (human face, Experiment 1) and in consuming (food products, Experiment 2) contexts.

First, we hypothesized that participants would spend more time in gazing at the SL than at the FL. This hypothesis is based on the previous finding suggesting that the FL involves only initial encoding of the current item, whereas the SL involves both encoding of the current item and comparison with the representation of the previous item [16]. Our experiments supported this hypothesis with the result that participants spent more time on the SL than on the FL, regardless of the type of decision task (i.e., preference vs. perceptual). Taken together, the data suggested that the two types of first dwell in 2AFC, i.e., the FL and the SL, may involve qualitatively different stages of decision making.

Second, we hypothesized that the gaze bias generated by selective encoding would be found only in the SL and not in the FL. This hypothesis is based on the assumption that until the time of the SL, participants cannot compare the items and do not know which one of the items is more appropriate to choose. The hypothesis was supported in Experiment 1 and partially in Experiment 2 (the wine block); participants indeed looked longer at the eventually chosen items in the SL, but not in the FL. The previous studies by Schotter et al. [11, 12] had predicted that the participants look longer at chosen items also in the FL. However, our results did not fully support this prediction, in that participants actively engaged in selective encoding *only* after they came to know the identity of both items. In other words, selective encoding and gaze bias may occur in the early comparison stage (SL), but not in the initial encoding stage (FL).

These results supported our modified model of selective encoding in the 2AFC paradigm (Fig 2). In the beginning of a trial, the participants look at an item at first (in FL). At this stage, the participants do not know which one of the items they should choose, and thus they only encode the current item (i.e., initial encoding stage). Next, the participants switch their gaze to another item (in the SL). At this stage, the participants have come to be able to compare the current item and the memory of the prior item (i.e., encoding and comparison stage). When the current item is considered better than the previous item, the participants allocate more time to the further encoding of the item that they are looking at; the gaze bias in SL. On the other hand, when the current item is not considered as the better choice, the participants cease looking at the item immediately and switch their gaze to the previous item; no gaze bias in the first dwells (i.e., FL nor SL). Therefore, the first dwell gaze bias would occur only when the eventually chosen item is looked at in the SL.

Although the present findings supported our model, the study has some limitations that need to be considered. First, the 2AFC paradigm mostly involves choice under conflict when the paired items are very closely matched in terms of the decision-making criteria [4–6], but the present study did not perform the matching manipulation. This is problematic because choice without conflict leads to less need for further information sampling [27]. Second, the variation of items was very low, and items were presented repeatedly through the experiment. This is also problematic because sampling behavior changes by previous encounters with the items [28] and participants could utilize prior knowledge about the items in their decision-making task [25, 26]. Familiarity and prior knowledge can also lead to less need for further information sampling. These two limitations potentially attenuate the selective encoding and gaze bias, and this could explain why a significant gaze bias for the chosen item was not found in the snack block in Experiment 2, when well-known snack products were presented repeatedly. In accordance with this concern, the results of Experiment 2 showed that the participants’ sampling behavior became quicker as the participants became more familiar with the stimuli through the experiment. However, the results also showed that such familiarization within the experiment did not affect the selective encoding and the gaze bias, which was found in Experiment 1 and in the wine block in Experiment 2. Therefore, it is suggested that the failure of showing the gaze bias in the snack block was not simply due to the repetitive stimulus presentation, rather to the familiarity of the snack products which had been formed in the participants’ daily experiences. Hence, these points should be addressed in future through well-controlled studies, and we speculate that such investigations will further validate our findings.

The gaze bias effect has been discussed intensively with reference to preference formation [3]. For instance, Shimojo and his colleagues [4–6] demonstrated that gaze bias (i.e., gaze cascade effect) for an eventually chosen item exists only in the preference decision task. Then, they proposed that eye movements during the decision affect the preference formation through a positive-feedback loop of preferential looking [7] and mere exposure effect [8]. However, some studies failed to replicate the preference-specific gaze cascade effect for the chosen item [9, 10] and found this effect *both* in preference and non-preference (i.e., perceptual) decision tasks. The first dwell gaze bias was also found both in preference and non-preference decision tasks in previous studies [9–12] as well as in the present study. The gaze bias effect for a chosen item was found even when the participants were asked to choose an item they do *not* want [29]. These findings suggest that the gaze bias effect for eventually chosen items does not have a causal effect on preference formation, but it rather reflects a general aspect of visual decision making. The effect is supposed to be caused by the top-down control of visual attention to the most appropriate item for their current decision task.

One of the practically important questions about relationship between choice and eye movement is how we can predict participants’ choice at an earlier stage in the choice process. Based on previous findings [9–12], it is predicted that the item that was looked at longer during the first dwell would eventually be chosen. However, our findings suggest that this prediction is only partially correct; the order of the dwell itself (i.e., FL or SL) tends to determine the size of the first dwell more strongly than does the gaze bias, and the gaze bias for the eventually chosen item emerges only after participants come to compare the items in the choice set. Therefore, taking the order of the first dwells into account would be a more effective way for quick and correct prediction of choice by participants’ eye movement. Although the present study was not designed to test this speculation, it should be addressed in appropriately designed experiments in future.

While we asked participants to make 2AFCs in the present study, this is an unusual situation in their daily life (e.g., purchasing products in a supermarket). However, it has been shown that participants use mostly pair-wise comparisons within subsets (referred to as the consideration set) even when multiple items are presented simultaneously [3, 30]. Thus, our findings can be applied to the choice process within the binary consideration set. Further studies that investigate the point at which selective encoding starts in a set-up in which more than two items are presented simultaneously are theoretically and practically important for understanding the relationship between choice and eye movement.

## Conclusion

The present study investigated how the order in which the eventually chosen items were looked at modulates selective encoding and gaze bias. The results showed that first dwell gaze bias was found only when the eventually chosen item was looked at after another item, indicating that selective encoding occurs in the early comparison stage of choice. These findings have important implications for understanding and explaining the relationship between choice and eye movement.

## Supporting information

S1 Table Generalized linear mixed models fitting the first dwell time in Experiment 1. To examine the necessity of separating the first and second datasets, we performed the GLMM analysis recruiting “Dataset,” first set (0) and second set (1), as a fixed effect along with “Task,” “Choice,” and “Look”. However, there was no significant effect or interaction of “Dataset” on the first dwell time.
(XLSX)

S2 Table Generalized linear mixed models fitting the first dwell time in the wine block in Experiment 2. There was no significant effect or interaction of “Dataset” on the first dwell time.
(XLSX)

S3 Table Generalized linear mixed models fitting the first dwell time in the snack block in Experiment 2. There was no significant effect or interaction of “Dataset” on the first dwell time.
(XLSX)

S4 Table Mean of first dwell time calculated in each participant in Experiment 1. (XLSX)

S5 Table Numbers of obtained observations in each participant in Experiment 1. (XLSX)

S6 Table Mean of first dwell time calculated in each participant in Experiment 2 (wine block). (XLSX)

S7 Table Numbers of obtained observations in each participant in Experiment 2 (wine block). (XLSX)

S8 Table Mean of first dwell time calculated in each participant in Experiment 2 (snack block). (XLSX)

S9 Table Numbers of obtained observations in each participant in Experiment 2 (snack block). (XLSX)
